# Preparation and structural determination of four metabolites of senkyunolide I in rats using ultra performance liquid chromatography/quadrupole-time-of-flight tandem mass and nuclear magnetic resonance spectra

**DOI:** 10.1186/s12906-016-1472-7

**Published:** 2016-12-05

**Authors:** Qiang Ma, Cong Ma, Fei Wu, Yao-kun Xiong, Yi Feng, Shuang Liang

**Affiliations:** 1 Engineering Research Center of Modern Preparation of TCM, Ministry of Education, Shanghai University of Traditional Chinese Medicine, Room 5117, No. 1200 Cai Lun Road, Pudong District, Shanghai 201203 People’s Republic of China; 2 Key Laboratory of Modern Preparation of TCM, Ministry of Education, Jiangxi University of Traditional Chinese Medicine, Nanchang, 330004 People’s Republic of China

**Keywords:** Senkyunolide I, Metabolites, UPLC/Q-TOF-MS, NMR, In vivo

## Abstract

**Background:**

Senkyunolide I (SEI) is one of the most important bioactive phthalides of *Ligusticum chuanxiong* Hort. (Umbelliferae), a Traditional Chinese Medicine. Our previous studies suggested that it might be developed as a potential treatment for migraine.

**Methods:**

In this paper, we aimed to isolate and characterize the main metabolites of SEI after gavage feeding in rats. Their structures were identified precisely on the basis of nuclear magnetic resonance (NMR) spectroscopy and UPLC/Q-TOF-MS spectrometry. We also established the main metabolic pathways of SEI in rats.

**Results:**

Four metabolites (M1-M4) were isolated, for the first time, from bile samples of rats by preparative high-performance liquid chromatography. Their structures were determined as SEI-6S-O-*β*-D-glucuronide (M1), SEI-7S-O-*β*-D-glucuronide (M2), SEI-7S-S-glutathione (M3) and SEI-7R-S-glutathione (M4) on the basis of the molecular mass of the analytes, using ultra performance liquid chromatography/quadrupole-time-of-flight mass spectrometry and 1D and 2D NMR.

**Conclusions:**

The results demonstrated that glucuronide and glutathione conjugation were the major pathways of SEI metabolism in vivo, and the configuration at the 7th-position could be inverted during glutathione conjugation.

## Background

*Ligusticum chuanxiong* Hort. (Umbelliferae) is a traditional Chinese herbal medicine that has been used to treat cardio- and cerebro-vascular disorders, such as angina pectoris, stroke and migraine, in China for thousands of years [1]. The chemistry and pharmacological effects of chuanxiong have been well documented. Phthalides have been reported as the primary bioactive constituents in this herb. Various pharmacological activities of the main phthalides, such as ligustilide and senkyunolide A, have been revealed, and their contributions to the beneficial effects of the herb are supported by in vivo and in vitro studies [2–5].

**Fig. 1:**
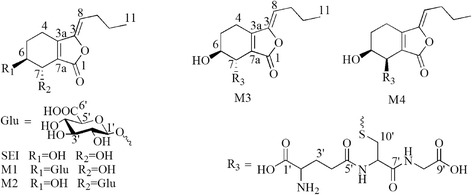
Chemical structures of the SEI and its metabolites M1–M4

Senkyunolide I (SEI, Fig. 1), a representative metabolite of ligustilide in vivo and in vitro [6, 7], is one of the most important bioactive constituents of chuanxiong [6, 8–11]. Several studies have demonstrated that SEI could decrease hydrogen peroxide (H_2_O_2_)-induced oxidative damage in cultured human liver HepG2 cells [7] and PC12 cells [12]. SEI could also decrease the morphological damage to red blood cells induced by concanavalin A [13]. Our previous study demonstrated that SEI could treat migraines, although the mechanism is unclear [14]. In addition, SEI is more stably in vitro, and has higher solubility [15, 16] and higher oral bioavailability [17, 18] compared with ligustilide and senkyunolide A. These properties of SEI suggest that it has a better potential as a new drug than ligustilide and senkyunolide A.

Generally, drug metabolism has a significant impact on the safety and efficacy of a drug and is commonly investigated at early stage of new drugs development. The identification of the drug’s metabolites is indispensable in this process [19]. Liquid chromatography (LC) coupled with tandem mass spectrometry has become a powerful tool to study drug metabolism because of its superior sensitivity and specificity [20, 21]. Quadrupole-time-of-flight mass spectrometry (Q/TOF-MS) is very useful in the characterization of drug metabolites because the technique provides accurate masses of ions and reveals valuable structural information [22]. In our previous study [23], 18 metabolites were characterized on the basis of ultra performance liquid chromatography/Q/TOF-MS (UPLC/Q-TOF-MS) analysis. However, because there are two chiral carbons (6-C and 7-C) in the molecular structure of SEI, the chemical structures of SEI conjugates present in vivo have not been fully characterized using Q/TOF-MS. To confirm further the structures of the main metabolites, and to obtain the pharmacological and toxicological information on them, it is essential to obtain adequate reference standards. Therefore, as part of our research on SEI, we aimed to isolate and characterize the metabolites of SEI, and to confirm the principal pathways of SEI metabolism in vivo.

In this study, four main metabolites were isolated from bile samples after gavage feeding of 100 mg/kg of SEI to rats. Their structures were identified precisely on the basis of nuclear magnetic resonance (NMR) and UPLC/Q-TOF-MS spectra. We also established the main metabolic pathways of SEI in rats.

## Methods

### Chemicals and reagents

SEI (purity >99.1%, as tested by HPLC-UV) was obtained from *L. chuanxiong* extracts in our laboratory. Its structure was confirmed by comparison of its MS and NMR profiles with that published in the literature [24, 25]. Slices of *L. chuanxiong* (No. 20130419) were purchased from Bozhou Medicinal Materials Company (Anhui Province, China) and authenticated by Professor Zhi-li Zhao of the Shanghai University of Traditional Chinese Medicine (Shanghai, China). The ultrahigh purified water used in this study was prepared in a Milli-Q water purification system (Millipore Corp., Billerica, MA, USA). Methanol and acetonitrile (HPLC grade) were purchased from Merck KGaA (Darmstadt, Germany). MeOH-d4, with tetramethylsilane (TMS) as internal standard for NMR analysis, was purchased from Cambridge Isotope Laboratories, Inc. (Andover, MS, USA). MCI Gel CHP 20P (75 μm–150 μm) for column chromatography was purchased from Mitsubishi (Tokyo, Japan). Sephadex LH-20 was obtained from GE Healthcare Bio-Sciences AB (Sweden). Other reagents and chemicals, including formic acid, were of analytical grade.

### Animals and drug administration

Twenty-eight male Sprague–Dawley rats (275–300 g body weight) were provided by the Experimental Animal Centre, Shanghai University of Traditional Chinese Medicine, China. Animals were housed at an ambient temperature of 24 ± 2 °C and 60 ± 5% humidity, with a 12 h dark–light cycle. They were kept in an environmentally controlled breeding room and given tap water and fed ad libitum until 12 h before the experiment. Animal experimental procedures and welfare were strictly in accordance with the Guide for the Care and Use of Laboratory Animals and the related ethics regulations of Shanghai University of TCM. Our animal protocol was approved by the Institutional Animal Care and Use Committee, Shanghai University of TCM (Shanghai, China). SEI was dissolved in deionized water (10 mg•ml-1) and administered by gavage at a dose of 100 mg•kg-1 body weight.

### Samples collection and processing procedures

The 28 rats were intraperitoneally injected with urethane (1.0 g · kg^−1^ body weight). Under anesthesia, a polyethylene cannula was inserted into the bile duct. Bile samples were collected for 12 h (approximately 400 mL in total) after oral administration of SEI at a dose of 100 mg · kg^−1^ body weight. Blank rat bile was collected before oral administration. All the samples were stored at −20 °C until further isolation and analysis.

Bile samples (100 μL) for HPLC-UV analysis were mixed with 400 μL of methanol for 60 s. They were then centrifuged at 13,000 rpm for 10 min, the supernatant was next transferred to a clear Eppendorf tube and then used 20 μLwas analysed using a HPLC-UV system.

### Isolation and purification of the metabolites of bile samples

About 400 ml of bile samples, which were centrifuged at 5,000 rpm for 10 min at 4 °C firstly, were subjected to MCI Gel CHP 20P column chromatography (Φ 4.5 × 70 cm) and eluted with a MeOH-H_2_O stepwise gradient (0:100, 10:90; 20:80, 30:70, 45:55, 60:40, v/v). The metabolites were mainly obtained from the 10%, 20% and 30% MeOH-H_2_O fractions. The fractions were concentrated in vacuo to yield residues. The residues were dissolved in a small amount of 30% MeOH-H_2_O, subjected to a Sephadex LH-20 column (Φ 2.5 × 150 cm) and eluted with 30% MeOH-H_2_O. The metabolites were further purified by preparative HPLC, performed with an Agilent Zorbax Eclipse XDB-C18 (5 μm, 9.4 × 250 mm) at 30 °C in an Agilent 1200 HPLC system (Agilent Technologies, USA). The detection wavelength was set at 278 nm. The mobile phase component A was water and B was acetonitrile, at a flow rate of 3 ml.min^−1^, and the column was eluted with a linear gradient of 4–15% B over 0–15 min, 15–20% B over 15–42 min, 20–60% B over 42–44 min and finally, the column was reconditioned with 4% B for 4 min to give M1 (57 mg), M2 (68 mg), M3 (14 mg) and M4 (61 mg).

### HPLC-UV analysis conditions

Analytical HPLC-UV analysis was carried out on an Agilent 1200 Series analytical HPLC system (Agilent Technologies, USA). A 20-μL injection loop and a Grace C18 reversed-phase column (5 μm, 4.6 × 150 mm), protected by an Security Guard Cartridges C18 (5 μm, 4 × 3.0 mm) guard column, were used for analysis. The analytic HPLC conditions comprised: a flow rate of 1 mL•min^-1^; the mobile phase component A was water with 0.1% formic acid and B was acetonitrile; the column was eluted with a linear gradient of 4% B over 0–2 min, 4–8% B over 2–5 min, 8–28% B over 5–41 min, 28–4% B over 41–43 min and the composition was maintained 4% B for 2 min. The detector wavelength was set at 278 nm and column temperature was maintained at 25 °C.

### Chromatographic and mass spectrometric conditions

Chromatography was performed on an ACQUITY™ UPLC system (Waters Corp., Milford, MA, USA) with a conditioned auto sampler at 10 °C. The chromatographic separation was carried out on an ACQUITY UPLC HSS T3 column (1.8 μm, i.d. 2.1 × 100 mm). The column temperature was maintained at 45 °C. The analysis was achieved with gradient elution using A (aqueous 0.1% formic acid) and B (acetonitrile) as the mobile phase. The gradient condition of the metabolites analysis was 0–15 min 20% B. The injection volume was 5 μL. The electrospray ionization source was operated in positive ionization mode with the capillary voltage at 2.7 kV. The source and desolvation temperatures were set to 120 °C and 400 °C, respectively. The cone gas flow was 50 L.h^−1^ and the desolvation gas was set to 700 L/h.

### NMR spectroscopy

A Broker AV 600 NMR spectrometer (Faellanden, Switzerland) was used to record 1H NMR (600 MHz) and 13C NMR (125 MHz) spectra in C5D5N at 25 °C. Chemical shifts were expressed in parts per million (ppm), with tetramethylsilane as the standard.

## Results

### Isolation and identification of SEI metabolites

**Fig. 2:**
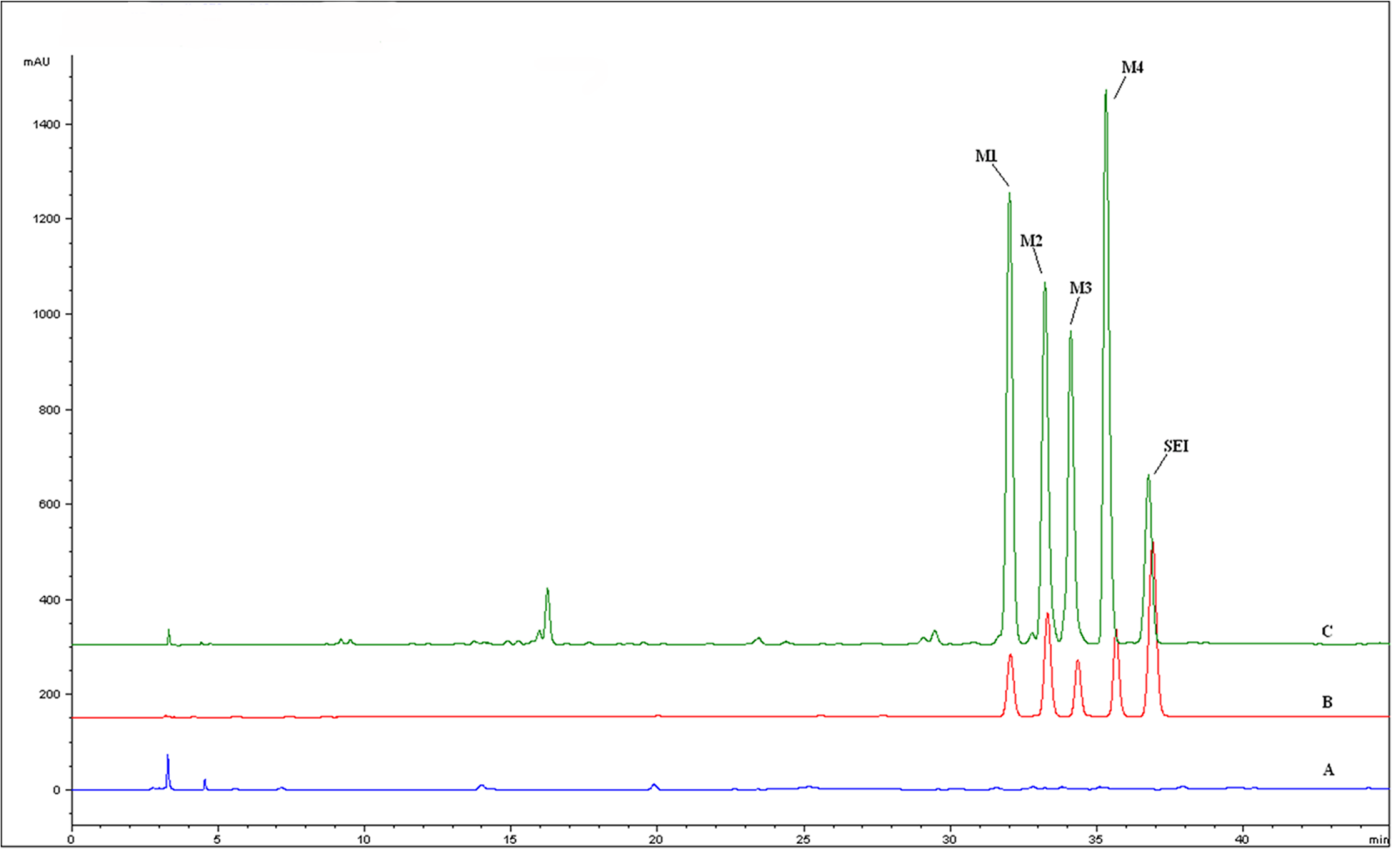
Chromatograms for the analysis of the four metabolites of SEI in rat bile. (A) HPLC-UV chromatograms of control bile; (B) HPLC-UV chromatograms of M1-M4 and SEI standards without bile; (C) Bile samples from rats after oral administration 100 mg/ml of SEI

As shown in Fig. 2, four main metabolites were found in bile samples of rats after oral administration of SEI, none of which had been isolated previously. The chemical structures of the metabolites were elucidated on the basis of UPLC/Q-TOF-MS and NMR spectra (^1^H NMR, ^13^C NMR), heteronuclear single quantum coherence (HSQC) and the heteronuclear multiple-bond correlation (HMBC).

**Fig. 3:**
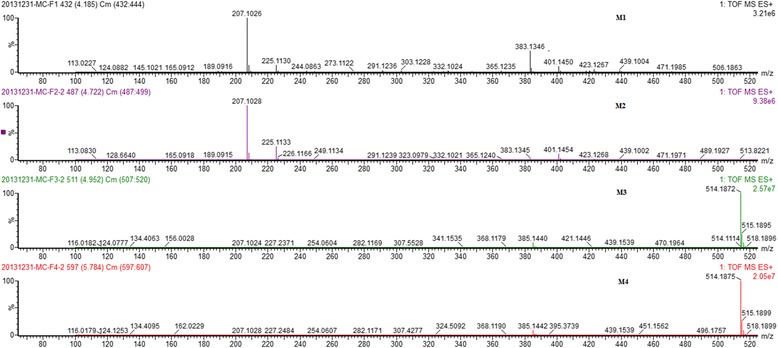
MS spectra of M1-M4

Careful analysis of the data collected from the UPLC/Q-TOF-MS system resulted in the identification of four metabolites in rat bile (M1-M4). Their [M + H]^+^ ions were at m/z 401.1455, 401.1453, 514.1870 and 514.1874 for M1-M4, respectively (Fig. 3). The elemental compositions, experimental mass, calculated mass, double-bond equivalents (DBE) and the mass errors of the [M + H]^+^ ions are shown in Table 1. The λ max values observed in the UV spectra of these four metabolites were 274 (M1), 275 (M2), 278 (M3) and 277 (M4) nm, suggesting that the metabolites have similar chromophore frameworks to SEI. The structures of theses metabolites were further elucidated by NMR spectroscopy. Metabolites M1 and M2 were glucuronide conjugates, and the linkage positions were at the 6- or 7-hydroxyl positions. For the glutathione conjugates metabolites M3 and M4, the linkage positions were at the 7-hydroxyl position, but they are cis-trans isomers of each other.

**Table 1:** The mass data for metabolites of Senkyunolide I (SEI)

Compound	Observed mass (Da)	Calculated mass (Da)	Error (mDa)	Error (ppm)	DBE	Norm	Formula
M1	401.1455	401.1448	0.6	1.5	6.5	0.048	C_18_H_25_O_10_
M2	401.1453	401.1448	0.7	1.7	6.5	0.004	C_18_H_25_O_10_
M3	514.1870	514.1859	1.4	2.7	8.5	2.062	C_22_H_32_N_3_O_9_S
M4	514.1874	514.1859	1.5	2.9	8.5	1.554	C_22_H_32_N_3_O_9_S

### Metabolites M1 and M2

**Fig. 4:**
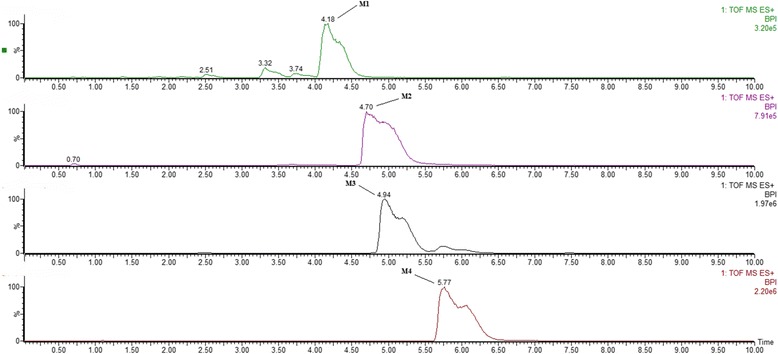
Representative UPLC/MS chromatograms of M1-M4

Metabolites M1 and M2 showed retention times of 4.18 and 4.70 min on the UPLC system (Fig. 4), with the same protonated molecular ion at *m/z* 401. Q-TOF-MS showed the protonated molecular ion at *m*/*z* 401.1455 (calculated 401.1448) for M1 and 401.1453 (calculated 401.1448) for M2, corresponding to the same molecular formula, C_18_H_25_O_10_. The molecular formulas of the two metabolites were further supported by the NMR spectral data.

The ^13^C NMR data of M1 (Table 2) clearly showed the existence of a glucuronic acid unit [*δ*_C_ 104.2 (C-1′), 74.8 (C-2′), 77.9 (C-3′), 73.6 (C-4′), 76.4 (C-5′) and 176.8 (C-6′)] [26]. It also showed signals from the butylidene side chain in the ^13^C NMR data [*δ*_C_ 114.4 (C-8), 29.0 (C-9), 23.3 (C-10) and 14.1 (C-11)] in the aglucon part of M1. The ^13^C NMR spectra [*δ*_C_ 170.8 (C-1), 150 (C-3), 156.0 (C-3a) and 126.4 (C-7a)] certified a five membered lactone ring. Two oxygen-bearing carbon signals were observed at *δ*_C_ 79.5 (C-6) and 63.5 (C-7), which were both tertiary carbons and the vicinal hydroxymethine protons appeared at *δ*_H_ 4.07 (m) and 4.46 (d, *J* = 3.3 Hz). All the NMR spectra of M1 were similar to those of SEI [24, 25], except for the additional glucuronic acid unit. In our previous study [23], we noted that glucuronidation was one of the major metabolic pathways of SEI in vivo, and the structure this metabolite was elucidated as SEI-6S-O-*β*-D-glucuronide or SEI-7S-O-*β*-D-glucuronide. In the present study, there was a long-range correlation between H-6 (*δ*_H_ 4.07, m) and C-1′ (*δ*_C_ 79.5) in HMBC spectrum (Table 2). Thus, the structure of M1 was definitively identified as SEI-6S-O-*β*-D-glucuronide with the help of 2D-NMR.

**Table 2:** 1D and 2D nuclear magenetic resonance (NMR) data of metabolites M1 and M2. (δ in ppm, MeOH-*d*_4_)

Position		M1		M2		
	δ_C_	δ_H_	HMBC	δ_C_	δ_H_	HMBC
1	170.8	-	-	171.0	-	-
3	150.0	-	-	150.0	-	-
3a	156.0	-	-	156.1	-	-
4	18.7	2.48 (1H, ddd, *J* = 18.7, 5.6, 3.4 Hz)	C-5, 6, 7a	18.8	2.53 (2H, m)	C-3a, 5, 6, 7a
		2.67 (1H, ddd, *J* = 18.2, 9.5, 5.8 Hz)	C-3a, 5, 6, 7a			
5	24.3	2.01 (1H, m)	C-3a, 4	26.2	1.90 (1H, m)	C-3a, 4, 6, 7
		2.18 (1H, ddd, *J* = 14.0, 8.9, 5.4 Hz)	C-3a, 4, 6, 7		2.01 (1H, dt, *J* = 13.7, 5.7 Hz)	C-3a, 4, 6, 7
6	79.5	4.07 (1H, m)	C-4, 7, 7a, 1′	70.2	4.13 (1H, m)	C-4, 5, 7, 7a
7	63.5	4.46 (1H, d, *J* = 3.3 Hz)	C-1, 3a, 5, 6, 7a	74.9	4.44 (1H, d, *J* = 3.9 Hz)	C-1, 3a, 5, 6, 7a, 1′
7a	126.4	-	-	125.3	-	-
8	114.4	5.45 (1H, t, *J* = 7.9 Hz)	C-3, 3a, 9, 10	115.2	5.48 (1H, t, *J* = 7.9 Hz)	C-3, 3a, 10
9	29.0	2.34 (2H, dd, *J* = 15.0, 7.6 Hz)	C-3, 8, 10, 11	29.1	2.34 (2H, dd, *J* = 15.0, 7.5 Hz)	C-3, 8, 10, 11
10	23.3	1.51 (2H, m)	C-8, 9, 11	23.3	1.52 (2H, dt, *J* = 14.7, 7.3 Hz)	C-8, 9, 11
11	14.1	0.96 (3H, t, *J* = 7.4 Hz)	C-9, 10	14.1	0.95 (3H, t, *J* = 7.4 Hz)	C-9, 10
1′	104.2	4.46 (1H, d, *J* = 7.8 Hz)	C-3′, 5′	104.7	4.68 (1H, d, *J* = 7.8 Hz)	C-7
2′	74.8	3.14 (1H, t, *J* = 8.3 Hz)	C-1′, 3′	75.1	3.20 (1H, t, *J* = 8.4 Hz)	C-1′, 3′
3′	77.9	3.38 (1H, t, *J* = 8.9 Hz)	C-2′, 4′	77.8	3.42 (1H, t, *J* = 6.0 Hz)	C-4′
4′	73.6	3.41 (1H, t, *J* = 9.1 Hz)	C-3′, 6′	73.6	3.44 (1H, t, *J* = 5.8 Hz)	C-3′, 6′
5′	76.4	3.57 (1H, d, *J* = 9.4 Hz)	C-1′, 3′, 4′, 6′	75.8	3.63 (1H, m)	C-1′, 3′, 4′, 6′
6′	176.8	-	-	176.8	-	-

The ^13^C NMR data of M2 (Table 2) also clearly showed the existence of a glucuronic acid moiety [*δ*_C_ 104.7 (C-1′), 75.1 (C-2′), 77.8 (C-3′), 73.6 (C-4′), 75.8 (C-5′) and 176.8 (C-6′)]. The ^13^C NMR data of the aglycone part of M2 (Table 2) were similar to those of M1 (Table 2), except for the carbon signals of C-6 (*δ*_C_ 70.2) and C-7 (*δ*_C_ 74.9), which were different from those of M1. Based on the cross peak between C-1′ (*δ*_C_ 104.7) and H-7 (*δ*_H_ 4.44, d, *J* = 3.9 Hz) in the HMBC spectrum, the structure of M2 was elucidated as SEI-7S-O-*β*-D-glucuronide.

### Metabolites M3 and M4

Metabolites M3 and M4 showed retention times of 4.94 and 5.77 min on the UPLC system (Fig. 4), with the same protonated molecular ion at *m*/*z* 514. Q-TOF-MS showed the protonated molecular ion at *m*/*z* 514.1870 (calculated 514.1859) for M3 and 514.1874 (calculated 514.1859) for M4, corresponding to the same molecular formula, C_22_H_32_N_3_O_9_S. The molecular formulae of the two metabolites were further supported by the NMR spectral data.

The assignments of all the proton and carbon signals for M3 and M4 were achieved by 1D and 2D NMR experiments, and the data are listed in Table 3. The ^13^C NMR data of M3 clearly showed a glutathione unit [*δ*_C_ 174.2 (C-1′), 55.8 (C-2′), 33.3 (C-3′), 27.8 (C-4′), 175.6 (C-5′), 55 (C-6′), 172.3 (C-7′), 44.6 (C-8′), 176.1 (C-9′) and 36.7 (C-10′)] [27]. The NMR data of the other C atoms suggested that M3 has the same parent construction as M1 and M2. In our previous study [23], we also observed that glutathione conjugation was one of the metabolic pathways of SEI in vivo. The HMBC spectra showed cross peaks between C-10′ (*δ*_C_36.7) and H-7 (*δ*_H_ 3.86, d, *J* = 3.3 Hz). Thus, M3 was characterized as SEI-7S-S-glutathione.

**Table 3:** 1D and 2D nuclear magenetic resonance (NMR) data of metabolites M3 and M4. (δ in ppm, MeOH-*d*_4_)

Position		M3		M4		
	δ_C_	δ_H_	HMBC	δ_C_	δ_H_	HMBC
1	169.9	-	-	170.8	-	-
3	149.7	-	-	150.0	-	-
3a	152.9	-	-	153.4	-	-
4	21.2	2.46 (1H, m)	C-3a, 5, 6, 7a	17.4	2.46 (1H, m)	C-3a, 5, 6, 7a
		2.57 (1H, m)	C- 3a, 6, 7a		2.50 (1H, m)	C-3a, 5, 7a
5	26.9	1.94 (2H, m)	C-3a, 4, 6, 7	24.6	1.95 (2H, m)	C- 3a, 4, 6, 7
6	70.3	4.08 (1H, dt, *J* = 11.2, 3.6 Hz)	C-4, 5, 7	70.0	4.17 (1H, m)	C-4, 7a
7	45.4	3.86 (1H, d, *J* = 3.8 Hz)	C-1, 3a, 5, 6, 7a, 10′	43.1	3.59 (1H, s)	C-1, 3a, 5, 6, 7a, 10′
7a	127.3	-	-	125.7	-	-
8	114.5	5.37 (1H, t, *J* = 7.9 Hz)	C-3, 3a, 10	114.2	5.40 (1H, t, *J* = 7.9 Hz)	C-3, 3a, 10
9	29.1	2.29 (2H, dd, *J* = 15.0, 7.6 Hz)	C-3, 8, 10, 11	29.0	2.31 (2H, dd, *J* = 15.0, 7.5 Hz)	C-3, 8, 10, 11
10	23.3	1.48 (2H, dd, *J* = 14.7, 7.4 Hz)	C-8, 9, 11	23.3	1.48 (2H, m)	C-8, 9, 11
11	14.1	0.93 (3H, m)	C-9, 10	14.1	0.93 (3H, t, *J* = 7.4 Hz)	C-9, 10
1′	174.2	-	-	174.5	-	-
2′	55.8	3.63 (1H, m)	C-1′, 3′, 5′	55.7	3.63 (1H, t, *J* = 6 Hz)	C-1′, 3′, 5′
3′	33.3	2.60 (2H, m)	C-2′, 4′, 5′	33.2	2.54 (2H, m)	C-2′, 4′, 5′
4′	27.8	2.15 (2H, dt, *J* = 14.3, 7.3 Hz)	C-2′, 3′, 5′	28.0	2.13 (2H, m)	C-2′, 3′, 5′
5′	175.6	-	-	175.4	-	-
6′	55.0	4.67 (1H, m)	C-5′, 7′, 10′	55.0	4.66 (1, m)	C-5′, 7′, 10′
7′	172.3	-	-	172.2	-	-
8′	44.6	3.72 (2H, s)	C-7′, 9′	44.7	3.69 (1H, d, *J* = 17.1 Hz)	C-7′, 9′
					3.77 (1H, d, *J* = 17.1 Hz)	C-7′, 9′
9′	176.1	-	-	176.4	-	-
10′	36.7	3.12 (1H, dd, *J* = 14.2, 7.7 Hz)	C-7, 6′, 7′	35.7	3.00 (1H, dd, *J* = 13.9, 8.4 Hz)	C-7, 6′, 7′
		3.22 (1H, dd, *J* = 14.1, 5.0 Hz)	C-7, 6′, 7′		3.22 (1H, dd, *J* = 13.9, 5.3 Hz)	C-7, 7′

The ^1^H NMR and ^13^C NMR data of M4 were similar to those of M3, and the same planar structure as that of M3 was deduced from the HSQC and HMBC data of M4, which suggested that M4 is a diastereoisomer of M3. In the ^1^H NMR, the H-7 of M4 (*δ*_H_ 3.59, s) was a single peak, different from H-7 (*δ*_H_ 3.86, d, *J* = 3.6 Hz) in M3, which suggested that M4 is in the 6, 7-cis-configuration [28]. Thus, the configurations of the 7-substituted groups were inverted during the metabolic process, and M4 was identified as SEI-7R-S-glutathione.

## Discussion

In our previous study [18], SEI was rapidly absorbed and exhibited extensive distribution after oral administration. Its oral bioavailability was approximately 37.25%, but it was quickly eliminated from plasma, with a half life of less than 60 min. In a second metabolic experiment [23], the biotransformation of SEI was investigated using UPLC/Q-TOF-MS. Eighteen metabolites were identified and the result indicated that methylation, hydration, epoxidation, glucuronidation and glutathione conjugation were the major pathways of SEI metabolism in vivo. Based on the results above, the fast elimination from plasma could be explained by rapid and extensive metabolism. However, the chemical structures of SEI conjugates present in vivo have not been fully characterized.

**Fig. 5:**
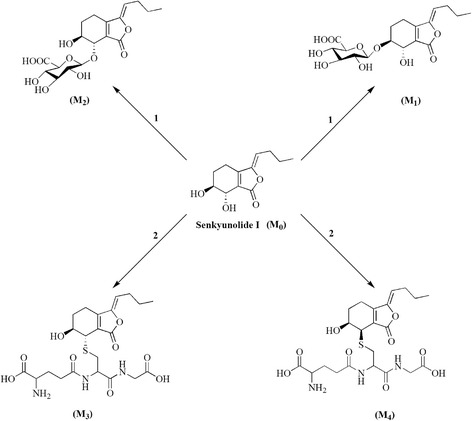
The major metabolic pathways of SEI in rat bile samples after oral administration of 100 mg/kg SEI, including glucuronidation (1) and glutathione conjugation (2)

In the present study, for the four main metabolites in bile samples, an elementary conclusion could be gained by analyzing the height and area of the peaks in HPLC-UV chromatograms. It suggested that SEI-6S-O-*β*-D-glucuronide (M1), SEI-7S-O-*β*-D-glucuronide (M2), SEI-7S-S-glutathione (M3) and SEI-7R-S-glutathione (M4) were the major metabolites in vivo. In our experiment, only trace metabolites of SEI were determined in the urine of rats, all of which suggested that the final excretion pathway in rats was bile. The metabolic pathways of SEI in rat bile mainly involved glucuronidation and glutathione conjugation during the phase II biotransformation pathway in rats because of the 6- and 7-hydroxyl groups in its structure, and the configurations of 7-substituted groups may be inverted during glutathione conjugation. Based on the structures of these metabolites, the proposed metabolic pathways of SEI are shown in Fig. 5.

Glutathione plays an important role in maintaining the redox state of cells via scavenging reactive oxygen species. Glutathione conjugation of xenobiotics is a detoxification pathway that inactivates electrophiles, which may covalently bind to endogenous proteins, resulting in potential detrimental effects [29]. This metabolic mechanism of SEI is indicated for its toxicity, and further work is needed to clarify the potential toxicity of SEI. In theory, medicines or metabolites are supposed to be excreted ultimately out of the body via faeces after bile excretion. However, we did not detect any metabolites, except for SEI, in fecal samples. Therefore, we suspect that the toxicity might be derived from its metabolites to a great extent. There are a few examples where phase II metabolism will produce significantly more biologically active or more toxic metabolites, including morphine [30] and certain heterocyclic aromatic amine compounds [31, 32]. In this study, we identified only four main metabolites of SEI in vivo and further research will be carried out to identify other metabolites. Issues to be addressed comprise structure–activity relationship analysis, bioactivity evaluation, and the quantitative mass balance of these metabolites.

## Conclusions

Our results demonstrated that glucuronide and glutathione conjugation are the major pathways of SEI metabolism in vivo, and the configuration at the 7th-position could be inverted during glutathione conjugation.

## Abbreviations

DBE: Double-bond equivalents
HMBC: Heteronuclear multiple-bond correlation
HSQC: Heteronuclear single quantum coherence
LC: Liquid chromatography
NMR: Nuclear magnetic resonance
Q/TOF-MS: Quadrupole-time-of-flight mass spectrometry
SEI: Senkyunolide I
TMS: Tetramethylsilane
UPLC/Q-TOF-MS: Ultra performance liquid chromatography/Q/TOF-MS
